# Angle-Resolved Enhancement of Rh800 Dye Photoluminescence Using Plasmonic Metal Gratings

**DOI:** 10.3390/ma19102141

**Published:** 2026-05-20

**Authors:** Aibibula Abudula, Paiziliya Maitiaximu

**Affiliations:** Xinjiang Key Laboratory of Hetian Characteristic Traditional Chinese Medicine Research, Xinjiang Hetian College, Hetian 848000, China; pazilya410@163.com

**Keywords:** surface plasmon resonance, metal grating, photoluminescence enhancement, Rh800 dye, angle-resolved spectroscopy, plasmonic biosensor

## Abstract

**Highlights:**

- Plasmonic metal gratings enhance Rh800 dye photoluminescence by 22-fold through resonant mode matching.
- FDTD simulations reveal hybrid plasmon-waveguide modes that confine the electric field at the PMMA-air interface.
- Angle-resolved spectroscopy enables quantitative assessment of PL enhancement across distinct excitation–collection configurations.
- Shallow grating geometry balances high enhancement with broad resonance tolerance and reproducibility.
- Cost-effective holographic lithography fabrication suits scalable biosensing and optoelectronic applications.

**Abstract:**

In this study, metal gratings were used to resonantly control and enhance the photoluminescence (PL) of Rh800 dye molecules at different angles by designing plasmon resonance modes that align with the excitation wavelengths. The experimental results show that when the plasmon resonance mode closely matched the excitation wavelength, the PL increased by a factor of 22. When the resonant position was not closely aligned with the excitation wavelength, PL enhancement remained significant, up to 14 times. Furthermore, even in the absence of a grating and with only a thin-film structure, the PL enhancement can still reach up to 5 times. Numerical simulations were also performed to analyze the effects of resonance mode, field distribution, and local field enhancement on the characteristics of the grating structure. The simulated results showed good agreement with experimental observations. The proposed structures offer greater flexibility for resonantly manipulating and enhancing PL across a wide range of applications. It offers significant opportunities in sensing, imaging, optoelectronics, energy conversion, catalysis, and biomedical diagnostics, such as disease biomarker detection.

## 1. Introduction

Plasmon-enhanced fluorescence (PEF) has become an effective approach for increasing molecular emission by utilizing the localized electromagnetic field enhancement produced by surface plasmon resonance (SPR) [1,2,3,4]. Among different plasmonic architectures, metallic gratings are particularly promising because they can couple incident light into surface plasmon polaritons (SPPs) through momentum matching, generating intense tunable near-fields at the metal-dielectric interface [5,6]. Compared with random nanoparticle arrangements or complex photonic crystals, gratings provide precise control over spectral and angular properties, which makes them highly suitable for directional emission and sensing applications [7,8,9]. However, most grating-based PEF studies have focused on visible-wavelength dyes. In contrast, the near-infrared (NIR) region, which is important for biomedical imaging and deep-tissue applications, has received comparatively less attention [3,10].

**Figure 1:**
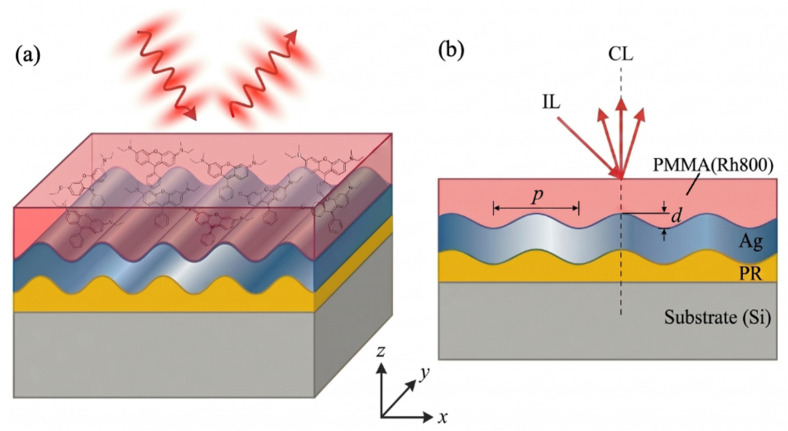
(**a**) 3D schematic view of the proposed Ag grating structure combined with Rh800 dye molecules. (**b**) 2D cross-sectional view of the structure. IL: Incident light. CL: Collected light.

Early pioneering work by Gómez Rivas et al. [5] showed that metallic gratings can control dye emission through SPP coupling. However, the reported enhancement factors were limited, and the angular dependence was not thoroughly analyzed. Later, Jiang et al. [6] demonstrated enhanced fluorescence of Rhodamine B on Ag grating films under normal incidence and achieved significant emission amplification. However, their study primarily used a single excitation and collection geometry, without clearly separating the effects of excitation enhancement from outcoupling contributions. More recently, Angelini and co-workers [7,8,9] explained the coupling mechanisms between plasmonic modes and fluorescence emission in nanostructured gratings, with particular emphasis on the role of waveguide plasmon hybridization. In contrast to these preceding studies, the present work focuses on the NIR dye Rh800 and establishes a custom angle-resolved spectroscopic platform that rigorously decouples excitation and collection pathways. It enables a clear and quantitative evaluation of enhancement factors under different angular and polarization conditions, which, to the best of our knowledge, has not been systematically addressed in prior grating-based PEF investigations. The proposed plasmonic–emissive hybrid structure, consisting of an Ag sinusoidal grating embedded beneath a PMMA(Rh800) capping layer, is schematically illustrated in Figure 1a,b.

**Figure 2:**
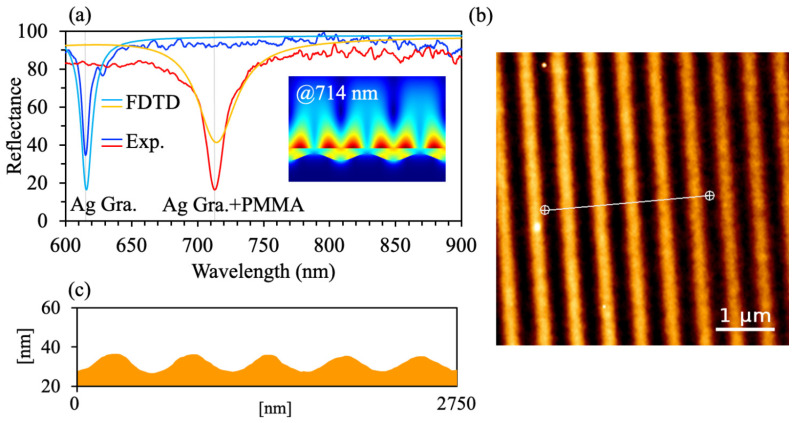
(**a**) Simulation and experimental resonance characteristics in reflection spectra of the grating structure periods of *p* = 540 nm under normal incidence of TM polarized light. The illustration depicts the distribution of the transverse electric field (|*E*|) at the resonance wavelengths of 714 nm. (**b**,**c**) AFM images of the as-fabricated structure. Exp: experimental. Gra: grating.

Rh800 is a near-infrared fluorophore with an absorption peak around 680–685 nm and emission peak centered near 710–730 nm [10,11,12]. Its spectral range falls within the biological NIR window of 650–900 nm, where tissue absorption and autofluorescence are reduced. It makes Rh800 highly suitable for in vitro and in vivo biosensing [3,10]. To achieve resonant enhancement of Rh800 excitation using a Ag grating, the grating period must satisfy the phase-matching condition for SPP excitation at the pump wavelength of 632.8 nm HeNe laser. At an oblique incidence angle of 40°, the grating equation predicts efficient coupling for grating periods close to 540 nm. Although the bare Ag grating shows a plasmon resonance at 610 to 620 nm, the PMMA (Rh800) overlayer causes a redshift and hybridization of this mode with the PMMA-air waveguide mode. As a result, a broadened resonance with full width at half maximum (FWHM) ≈ 60 nm is produced, which spectrally overlaps with the Rh800 emission band from 710 to 730 nm, as shown in Figure 2a. This trade-off relaxes nanofabrication tolerances while maintaining high enhancement [13,14,15].

In this study, a Ag sinusoidal grating with a period *p* = 540 nm and a shallow depth *d* ≈ 10–20 nm was used to systematically control and enhance the PL of Rh800 dye molecules at different angles. A custom angle-resolved spectroscopic platform was developed to independently control the excitation and collection pathways, allowing quantitative analysis of optical responses across different structural configurations. Finite-difference time-domain (FDTD) simulations were carried out to investigate the resonance behavior, field distribution, and local field enhancement, and the results were compared with experimental results. The results revealed that TM polarization produced a 22-fold PL enhancement under resonant spectral alignment, whereas TE polarization yielded only 4.1-fold enhancement—below the 5-fold Ag-film baseline—confirming the critical role of resonant plasmon coupling. Even under spectral mismatch, the enhancement remains significant at 14-fold. Furthermore, a continuous Ag film without grating patterning yields a 5-fold enhancement, underscoring the critical role of grating-coupled plasmon resonance. By elucidating the fundamental physical mechanisms and determining optimal operating conditions, this study improves the understanding of plasmon-enhanced NIR fluorescence. It also provides a flexible platform for the development of high-performance optical sensors, directional light-emitting devices, and biomedical diagnostic systems.

## 2. Structure and Experiments

### 2.1. Experimental Materials and Reagents

The following materials were used in this study. Rhodamine 800 (Rh800) has the molecular formula C_26_H_26_ClN_3_O_5_, a molecular weight of 495.95 g·mol^−1^, and CAS No. 137993-41-0 (Sigma-Aldrich, St. Louis, MO, USA, product code: 101951412). Poly (methyl methacrylate) (PMMA) has a nominal molecular weight of 996,000 g·mol^−1^ and CAS No. 9011-14-7 (Sigma-Aldrich, St. Louis, MO, USA, product code: 1001951940). The solvents and other reagents used were benzyl alcohol (≥99.5%, analytical grade), acetone (≥99.5%, analytical grade), anhydrous ethanol (≥99.8%, analytical grade), and deionized water with resistivity ≥ 18.2 MΩ·cm, obtained from a Milli-Q^®^ purification system (MilliporeSigma, Burlington, MA, USA). All reagents were used as received without additional purification.

### 2.2. Sample Preparation

Substrate cleaning: Standard commercial silicon wafers with p-type conductivity, 100-oriented, resistivity of 1 to 10 Ω·cm, and thickness of 525 ± 25 μm were cleaned using the standard RCA protocol. The wafers were ultrasonically cleaned for 15 min each in acetone, anhydrous ethanol, and deionized water to remove organic residues and particles, followed by drying with high-purity nitrogen gas. Photoresist grating fabrication: A periodic photoresist grating template was prepared using two-beam interference holographic lithography. A uniform layer of positive-tone photoresist (S1805, Shipley, Marlborough, MA, USA) was spin-coated onto the cleaned silicon substrate at 4000 rpm for 60 s, producing a film thickness of ~120 nm. The interference pattern was created using a coherent He–Cd laser with a wavelength *λ* = 325 nm and an exposure time of 30 s, corresponding to a fluence ≈ 15 mJ·cm^−2^. The exposed wafers were then developed in MF-319 developer (Dow Electronic Materials, Marlborough, MA, USA) for 6 to 8 s at 23 ± 1 °C. The resulting photoresist gratings exhibited a uniform period of *p* ≈ 540 nm, a shallow groove depth of *d* ≈ 10–20 nm, and root-mean-square (RMS) surface roughness < 0.5 nm, as confirmed by atomic force microscopy (AFM) characterization (Figure 2b,c). Metal grating deposition was done as follows. A continuous Ag film, ~150 nm thick, was deposited onto the photoresist grating by thermal evaporation under high vacuum with a base pressure < 5 × 10^−4^ Pa using a vacuum thermal evaporator (VZZ-300, VNANO, Beijing, China). The deposition rate was maintained at 0.2 ± 0.05 nm·s^−1^, monitored in real time using a quartz crystal microbalance (QCM) by tracking the oscillation frequency change in the vacuum chamber. The lift-off process was not performed, allowing the Ag grating to replicate the surface profile of the underlying photoresist structure. PMMA (Rh800) functional layer deposition: A homogeneous PMMA (Rh800) composite solution was prepared by dissolving 6.0 mg of Rh800 powder into 3.0 mL of a 5 wt% PMMA solution in benzyl alcohol. The mixture was sonicated for 60 min in a water bath at room temperature until complete dissolution was achieved, resulting in a homogeneous deep-blue solution. The Rh800 concentration in the solution was 2.0 mg·mL^−1^, corresponding to 4.0 × 10^−3^ mol·L^−1^. The prepared solution was spin-coated onto the Ag grating substrate at 3500 rpm for 60 s, followed by soft baking on a hotplate at 80 °C for 5 min to remove residual solvent and enhance film cohesion. The fabricated structure formed a plasmonic emissive hybrid system consistent with the design shown in Figure 1a,b.

**Figure 3:**
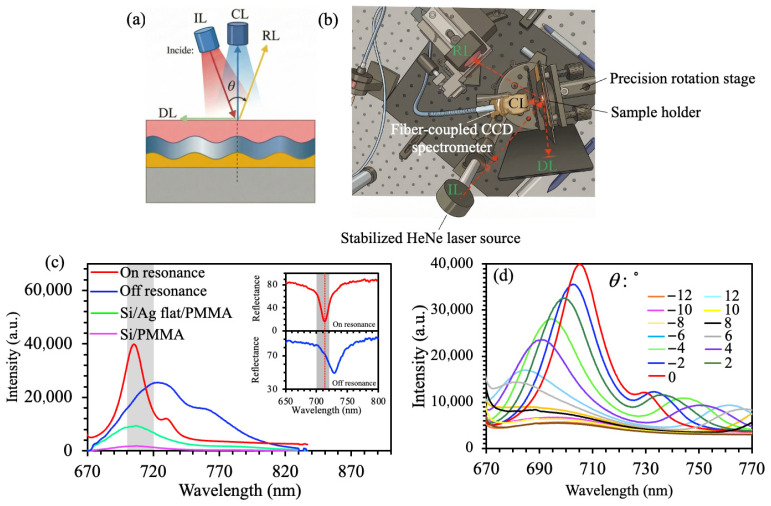
(**a**) Schematic illustration of the emission collection measurement for the plasmonic metal grating structure. IL: Incident light; CL: Collected light; RL: Reflected light; DL: Diffracted light. (**b**) Experimental setup for luminescence measurement. (**c**) Vertical PL spectra under 40° incidence (inset: Resonant and non-resonant reflection spectra). (**d**) Angle-resolved PL intensity in the resonant condition (incident angle: 40°; collection angle ranging from −12° to 12°).

The layer sequence and measurement geometries are schematically illustrated in Figure 1a,b. Figure 1a depicts the oblique-incidence (40°)/vertical-collection (0°) configuration, while Figure 1b shows the vertical-incidence (0°)/oblique-collection (40°) geometry. The PMMA (Rh800) capping layer had a uniform thickness of approximately 80 nm, as confirmed by stylus profilometry (Dektak XT, Bruker, Billerica, MA, USA). Based on the spin-coating process and the solubility characteristics of Rh800 in the PMMA-benzyl alcohol system, the Rh800 molecules are expected to segregate toward the upper region of the PMMA matrix during solvent evaporation. This distribution facilitates effective overlap with the enhanced near-field at the PMMA–air interface, as confirmed by the observed 22-fold PL enhancement (Figure 3c), which would be significantly weaker if the dye were uniformly dispersed throughout the ~80 nm PMMA layer.

### 2.3. FDTD Simulation Details

Numerical simulations based on the FDTD method were performed using Lumerical Solutions, version 2025R2, Vancouver, BC, Canada [16], to predict the resonance position and optimize the structural parameters before fabrication. Silver (Ag) was selected as the grating material because, within the operational wavelength range of this study (632–730 nm), it exhibits lower ohmic losses (smaller *ε*″) and higher SPP quality factors than Au or Al, ensuring more efficient field confinement and fluorescence enhancement for Rh800 [17]. This period was chosen to satisfy the phase-matching condition at 632.8 nm with an oblique incidence angle of 40°. After coating with the PMMA (Rh800) layer, the resonance of the bare grating shifted toward longer wavelengths, resulting in spectral overlap with the dye emission band, as shown in the simulated reflectivity in Figure 2a. As the metal grating layer is sufficiently thick (~150 nm) to prevent the transmission of incident light, the model does not consider the photoresist layer and silicon substrate. The simulation domain was divided using a non-uniform mesh with a minimum mesh size of 2 nm in the *z*-direction near the Ag grating ridges and 5 nm in the *x*-direction. Perfectly matched layer (PML) boundary conditions were applied at all domain boundaries to suppress artificial reflections. The total-field/scattered-field (TFSF) source was employed to excite TM-polarized plane waves at incident angles ranging from 0° to 60°. The dielectric properties of Ag were modeled using the experimental optical data reported in Palik’s Handbook of Optical Constants [17], while the refractive index of PMMA was fixed at *n* = 1.49. Frequency-domain field monitors were positioned at the PMMA air interface and near the Ag ridge apexes to capture the electric field intensity distribution, as shown in the inset of Figure 2a. Convergence was verified by ensuring that the reflected power stabilized within 0.5% across successive iterations.

### 2.4. Optical Characterization and Angle-Resolved PL Setup

Photoluminescence measurements were performed using a custom-built angle-resolved spectroscopic platform [8,9], as schematically shown in Figure 3a,b. The system was designed to clearly distinguish optical responses from different structural configurations by separating the excitation and collection pathways in real space, effectively removing spectral interference [18]. Figure 3a illustrates the fundamental measurement geometry, where Rh800 molecules were excited by oblique incidence light (IL) at an angle of 40° using a linearly polarized HeNe laser with a wavelength of *λ* = 632.8 nm and 5 mW. The emitted photoluminescence was collected only along the normal direction of the sample at 0°. Angular separation ensures that reflected light (RL) and diffracted light (DL) contributions fall outside the detection solid angle. For the grating period of *p* = 540 nm, the second-order diffraction (*m* = 2) of both the excitation light at 632.8 nm and the PL emission at 710 to 730 nm yields sinθ > 1 according to the grating equation. It indicates that the second-order diffracted waves are evanescent and cannot propagate into the far field. As a result, only the zeroth-order corresponding to specular reflection at 40° and the first-order diffracted light directed at ~32° from the surface normal can propagate in free space. Therefore, the angular separation between the excitation and collection arms completely prevents all propagating diffracted orders from the detection solid angle. Figure 3b presents the experimental architecture, which consisted of a stabilized HeNe laser source, a motorized half-wave plate for polarization control, a fiber-coupled CCD-based spectrometer (Ocean Optics QE Pro, Ocean Optics, Orlando, FL, USA) with an integration time of 100 ms, a spectral resolution of 1.2 nm, and a high-precision rotation stage with an angular resolution ≤ 0.5°. The rotation stage enabled continuous calibrated angular scanning over a range from −12° to +12°. The excitation intensity at the sample surface was approximately 4.0 mW·cm^−2^, determined from the incident laser power and the illuminated spot area. A long-pass filter (λ > 650 nm) was used to block scattered excitation light. Before the measurements were performed, the system was calibrated using a standard Lambertian scatterer to ensure angular accuracy within ±0.3°.

### 2.5. Enhancement Factor Quantification

The fluorescence enhancement was quantitatively evaluated using the established method of integrating hot-spot contributions [19]. The enhancement factor was determined by comparing the integrated fluorescence intensity with that of reference substrates [20], as applied to the spectra presented in Figure 3c. Spectral integration was performed over the wavelength range 675 to 850 nm, encompassing the entire Rh800 emission band, as shown in Figure 3c. Background subtraction was performed using a linear baseline fitted to the spectral regions near the emission peak. A bare Si wafer spin-coated with PMMA (Rh800) without a Ag layer was used as the intrinsic reference, corresponding to an enhancement factor EF = 1, blue curve, shown in Figure 3c. For the Ag thin-film control, a continuous 150 nm Ag layer deposited on Si was used (green curve in Figure 3c). All reported enhancement factors are averages over three independently fabricated samples, with error bars denoting standard deviation.

## 3. Results and Discussion

The spectral location of the SP resonance plays an important role in determining its overlap with the emission characteristics of the luminophore, since it strongly influences both the excitation absorption efficiency and the radiative emission process [21]. When the resonant position aligns with the luminescence peak, the system can efficaciously enhance the interaction between light and matter, thereby augmenting the intensity of fluorescence, phosphorescence, or other luminescence processes. Therefore, the resonance-matching conditions were investigated through experimental reflectivity measurements and FDTD theoretical simulations [22,23,24]. Figure 2 illustrates the resonance characteristics and structural profiles of a plasmonic structure, along with as-fabricated gratings with a period *p* = 540 nm. Figure 2b,c presents an AFM image of the fabricated structure. Figure 2a presents the reflectivity spectrum of a pure metal grating compared to a metal grating coated with a layer of dye molecule PMMA (Rh800). Theoretical calculations that correspond to these measurements are also provided. As shown in Figure 2a, the resonant positions of the experimental reflection spectra are highly consistent with the theoretical simulation results. However, the experimental reflectivity dip is slightly broader than the simulated curve, primarily due to finite grating length (~1 mm vs. infinite periodicity in FDTD), surface roughness (RMS ~0.5 nm, Figure 2b), and uncertainty in the tabulated Ag dielectric constants [17]. The resonant position agrees within ~5 nm (<1% error), confirming the model reliability. To explain the physical origin of resonance enhancement, FDTD simulations of the electric field intensity (|*E*|) distribution at the luminescence resonant wavelength λ = 714 nm was performed. These simulations integrate quantitative structural parameters derived from AFM. The inset of Figure 2a shows that the maximum electric field is mainly localized at the interface between the PMMA and the air on the upper surface. Furthermore, additional localized enhancement occurs at the contact region between the Ag grating ridges and the PMMA layer. Negligible field penetration into the bulk of the PMMA waveguide layer is observed. This spatial confinement shows that the resonance is primarily controlled by the dielectric waveguide mode supported at the PMMA–air interface. There is only a weak hybridization with the SP mode excited by the Ag grating. These factors together produce a mixed quasi-bound state that is both photonic and plasmonic in nature [15]. The enhanced electric field overlaps spatially with the Rh800 dye molecules, which are confined to the top ~10 nm of the PMMA layer. Because of this overlap, the efficiency of molecular excitation increases significantly. Figure 2b presents the AFM topography, revealing a highly periodic, uniform grating structure with a period *p* = 540 nm, ridge width *w* ≈ 270 nm, and root-mean-square (RMS) surface roughness < 0.5 nm, confirming excellent fabrication reproducibility. The cross-sectional AFM profile in Figure 2c quantifies a shallow grating depth *d* ≈ 10–20 nm and a sidewall angle ≈ 25°, indicating mild anisotropy in the etching process. The physical geometry inherently limits the SP–waveguide coupling strength, thereby reducing hybridization efficiency and broadening the resonance linewidth (FWHM). However, the resulting FWHM (~60 nm) aligns optimally with the intrinsic emission bandwidth of Rh800 dye. This spectral matching relaxes nanofabrication tolerances, enhances process robustness, and enables a 22 times increase in PL while significantly improving device-to-device reproducibility [13,14]. Now that the structural and resonant properties of the fabricated grating have been established, the next section examines the performance of the angle-resolved photoluminescence enhancement.

Figure 3c presents quantitative vertical PL spectra with a collection angle = 0° for three representative configurations. Firstly, the Si/PMMA substrate alone yields negligible emission with an integrated intensity ≈ 1814 counts. Secondly, deposition of a continuous Ag film enhances PL by 5.1-fold (integrated intensity: 9241 counts). Thirdly, patterning the Ag layer into a periodic grating yields further amplification, reaching 25,444 counts under partial resonance when the excitation wavelength is aligned with the plasmonic dispersion curve. The signal reaches a peak of ≈40,000 counts when both excitation and emission wavelengths coincide with the grating-coupled SPP resonance. This peak represents a 22-fold net enhancement compared to the bare substrate. The inset highlights the contrast between on-resonance and off-resonance emission profiles, underscoring spectral matching as the dominant factor governing the magnitude of enhancement. Figure 3d displays the angle-resolved PL intensity distribution under resonant excitation at an incident angle of 40°. On the negative angular side from –12° to 0°, it aligns with the direction of incident light, and the PL intensity is marginally higher than that on the positive side from 0° to +12°. It is likely attributable to residual scattering from the incident beam. The overall emission profile exhibits a narrow angular spread—characterized by a FWHM of approximately 8°—with its peak centered precisely at the normal direction (0°). Intensity decays monotonically with increasing collection angles, thereby demonstrating that the grating structure provides a high degree of control over emission directionality. Furthermore, weak tail features emerge in the long-wavelength spectral region beyond 800 nm. These features are attributed to residual first-order diffracted emission (*m* = −1) that is weakly scattered into the detection solid angle at large collection angles (>8°), as the grating partially outcouples SPP modes into non-normal directions. Their low intensity (<5% of the peak) confirms that the dominant emission is directed along the normal surface, consistent with the narrow angular spread (FWHM ~8°) shown in Figure 3d. Overall, these experimental results confirm the effectiveness of the resonant matching strategy between the grating-coupled SPP mode and the Rh800 excitation/emission bands, achieving a 22-fold enhancement in PL intensity under optimal TM-polarized, 40° oblique excitation. In addition, the results provide a strong experimental basis for the later numerical analysis of electromagnetic field distribution and local field enhancement.

**Figure 4:**
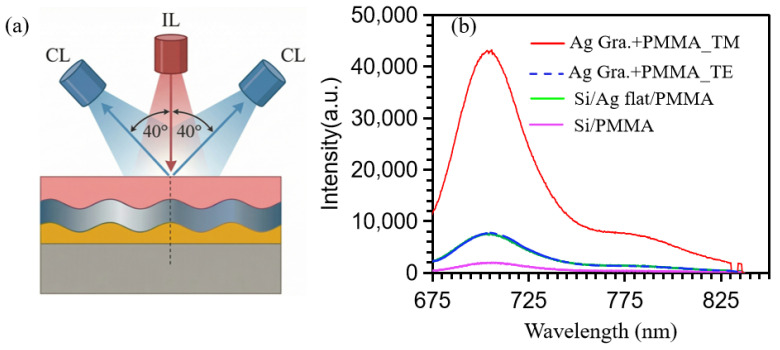
(**a**) Schematic of the plasmonic metal grating. (**b**) PL spectrum collected at 40° under normal incidence at resonance.

To further elucidate the polarization-dependent nature of the plasmonic enhancement, we investigated the PL response under TM- and TE-polarized excitation using complementary vertical-incidence geometry. Figure 4a illustrates the experimental geometry for PL detection under vertical optical excitation (0° incidence angle), with PL signals collected at a fixed angle of 40° relative to the normal surface. In this configuration, the Ag grating/PMMA (Rh800) hybrid structure was illuminated using either TE- or TM-polarized incident light to systematically investigate the polarization-dependent coupling between localized SP and Rh800 dye molecules, as well as its effect on PL enhancement. This setup complements the oblique-incidence and normal-collection geometry presented in Figure 3, enabling a comprehensive analysis of angular resolved optical responses under different excitation and collection configurations. Figure 4b displays the corresponding PL spectra, recorded over the wavelength range of 675 to 850 nm. All samples showed a dominant emission band in the range of 710 to 730 nm, which closely corresponds to the intrinsic fluorescence peak of Rh800. The spectral position and line shape of the dominant emission band confirm that the detected PL signal primarily originates from radiative recombination of the Rh800 dye molecules, with negligible contribution from other sources such as substrate fluorescence or plasmonic scattering. Quantitative analysis reveals that the bare Si/PMMA substrate yields the weakest PL intensity of ≈1900 a.u. The deposition of a continuous Ag film increased the PL signal by approximately 5 times to about ≈9500 a.u. In comparison, the patterned Ag grating exhibits strong polarization anisotropy. Under TM-polarized excitation, the PL intensity reaches ≈43,000 a.u., corresponding to a 22.6-fold enhancement compared with the Si/PMMA reference and a 4.5-fold enhancement relative to the Ag-film control. However, under TE-polarized excitation, the PL intensity was only ≈7800 a.u., representing a modest 4.1-fold enhancement over the substrate but falling slightly below the Ag-film baseline. This stark contrast originates from the fundamental symmetry requirements of plasmonic resonance. TM polarization enables efficient coupling to charge oscillations along the grating grooves, resulting in intense near-field localization that boosts both dye excitation and radiative decay rates. Similarly, TE polarization, whose electric field lies orthogonal to the grating periodicity, fails to drive resonant electron oscillations and thus affords only weak, non-resonant scattering-mediated enhancement. Collectively, these results unambiguously establish plasmon resonance as the primary physical mechanism governing PL enhancement in this system. Moreover, the results reveal that TM-polarized vertical excitation together with 40° off-normal collection provides the optimal operational condition. This finding offers a practical design strategy for developing high-efficiency plasmon-enhanced optoelectronic devices and ultrasensitive biosensors that operate under normal-incidence illumination. It is important to note that previous grating-based studies mainly investigated visible-wavelength dyes under fixed excitation geometries [6]. In contrast, the present angle-resolved investigation of NIR Rh800 reveals distinct polarization-dependent and angular-dependent behaviors that are not accessible through single-angle measurements. The shallow grating design, which intentionally trades coupling strength for spectral overlaps, represents a fundamentally different optimization strategy from that of deeper grating structures.

To contextualize the results within the field of grating-coupled plasmon-enhanced fluorescence, the shallow Ag sinusoidal grating is compared with representative experimental studies employing metal gratings and dye molecules, as shown in Table 1. Several important distinctions emerge from this comparison. First, the 22-fold resonant enhancement is competitive with established Ag-grating systems for visible dyes, such as 30× for R6G [6], and ~30× for optimized groove depth [25]. In addition, this study extends plasmon-enhanced fluorescence into the biological NIR window (Rh800, ~720 nm)—a spectral regime not addressed by prior grating studies [6,18,25,26,27]. Second, the shallow grating design (*d* ≈ 10–20 nm) in this work is consistent with the groove-depth optimization study [25], which identified ~20 nm as the depth that yields the maximal GC-SPR fluorescence enhancement. However, whereas [25] employed fixed-angle measurements, our angle-resolved platform systematically identifies the optimal 40° incidence/0° collection condition and reveals narrow directional emission (FWHM ~8°), providing quantitative angular discrimination inaccessible through single-angle measurements. Third, Tawa et al. [26,27] reported higher enhancement factors of 90–170× using Ag grating chips with optimized SiO_2_ spacer layers for Cy5 in the visible range. Although their spacer-optimization strategy maximizes EF, our single-layer PMMA (Rh800) coating achieves 22× enhancement through waveguide plasmon hybridization without additional dielectric spacers, offering a simpler fabrication route. The 5-fold enhancement from the continuous Ag film control confirms that the additional grating-induced improvement arises specifically from resonant SPP coupling and hybrid-mode confinement. Finally, Nicol & Knoll [18] reported modest 3–6× enhancement for grating-coupled SPCE, emphasizing directional emission control over intensity amplification. Our work bridges this gap by demonstrating both substantial enhancement (22×) and directional control (FWHM ~8°) within a unified angle-resolved architecture.

## 4. Conclusions

This study systematically explains the underlying mechanism responsible for the pronounced enhancement of Rh800 dye PL by plasmonic metal gratings, integrating rigorous theoretical modeling with experimental validation. FDTD simulations reveal that, under optimized grating parameters, TM-polarized light excites SPR, which forms a weakly coupled hybrid mode with the waveguide mode supported at the PMMA–air interface. This hybridization leads to strong confinement of the electric field, with maximum intensity localized at the PMMA–air interface and extending into the upper ~10 nm region of the PMMA layer where the Rh800 molecules reside. Secondary field enhancement occurs at the Ag ridge–PMMA contacts region. As a result, the localized field enhancement is significantly increased. Experimental results corroborate this mechanism. Under resonant TM-polarized excitation, the PL intensity increases by a factor of 22. Under partial resonance matching, the enhancement remains significant at 14-fold, surpassing the 5-fold enhancement achieved with a conventional planar Ag film. Although the shallow grating geometry inherently limits the strength of mode coupling, it simultaneously enhances phase-matching tolerance by broadening the resonance peak sufficiently to ensure robust spectral overlap with the wide emission band of the dye. This favorable trade-off thus reconciles high enhancement factors with excellent device reproducibility. The proposed structure was fabricated using holographic lithography and thermal evaporation, providing a simple, low-cost, and scalable fabrication process with excellent reproducibility between devices. The demonstrated capabilities, including 22-fold PL enhancement, narrow directional emission (FWHM ~8°), and strong TM-polarization selectivity, provide a foundational platform for plasmon-enhanced optoelectronic devices and directional light-emitting systems. The controllable angular emission and enhanced NIR fluorescence further suggest potential for integration into directional NIR emitters and plasmon-enhanced spectroscopic platforms. Overall, this work establishes a versatile technical platform that bridges fundamental plasmonic insight with practical application potential.

## Figures and Tables

**Table 1:** Comparison of fluorescence enhancement factors in selected metal-grating structures.

Ref.	Dye	Emission (nm)	Enhancement Factor
[6]	Rhodamine 6G	~580	>30×
[25]	Fluorescent dye	Visible	~30×
[18]	Organic dye layer	Varied	3–6×
[27]	Cy5-streptavidin	~670	90×
[26]	Cy5-streptavidin	~670	170×
This work	Rh800	~720	22× (resonant TM); 14× (partial); 5× (planar film)

## Data Availability

The original contributions presented in this study are included in the article. Further inquiries can be directed to the corresponding author.
